# Neutralization of Human Interleukin 23 by Multivalent Nanobodies Explained by the Structure of Cytokine–Nanobody Complex

**DOI:** 10.3389/fimmu.2017.00884

**Published:** 2017-08-21

**Authors:** Aline Desmyter, Silvia Spinelli, Carlo Boutton, Michael Saunders, Christophe Blachetot, Hans de Haard, Geertrui Denecker, Maarten Van Roy, Christian Cambillau, Heidi Rommelaere

**Affiliations:** ^1^Architecture et Fonction des Macromolécules Biologiques (AFMB), UMR 7257, Centre National de la Recherche Scientifique (CNRS), Marseille, France; ^2^Architecture et Fonction des Macromolécules Biologiques (AFMB), UMR 7257, Aix-Marseille Université, Marseille, France; ^3^Ablynx N.V., Ghent, Belgium

**Keywords:** interleukin 23, nanobody, multivalent binder, crystal structure, anti-inflammatory

## Abstract

The heterodimeric cytokine interleukin (IL) 23 comprises the IL12-shared p40 subunit and an IL23-specific subunit, p19. Together with IL12 and IL27, IL23 sits at the apex of the regulatory mechanisms shaping adaptive immune responses. IL23, together with IL17, plays an important role in the development of chronic inflammation and autoimmune inflammatory diseases. In this context, we generated monovalent antihuman IL23 variable heavy chain domain of llama heavy chain antibody (V_HH_) domains (Nanobodies^®^) with low nanomolar affinity for human interleukin (hIL) 23. The crystal structure of a quaternary complex assembling hIL23 and several nanobodies against p19 and p40 subunits allowed identification of distinct epitopes and enabled rational design of a multivalent IL23-specific blocking nanobody. Taking advantage of the ease of nanobody formatting, multivalent IL23 nanobodies were assembled with properly designed linkers flanking an antihuman serum albumin nanobody, with improved hIL23 neutralization capacity *in vitro* and *in vivo*, as compared to the monovalent nanobodies. These constructs with long exposure time are excellent candidates for further developments targeting Crohn’s disease, rheumatoid arthritis, and psoriasis.

## Introduction

By searching sequence databases for members of the IL6 cytokine family (1), a new protein, designated interleukin (IL) 23p19 was identified. This new protein had no biological activity, but formed in combination with the p40 subunit of IL12 a novel heterodimeric cytokine named IL23. The p40 subunit is shared with IL12, where it forms a heterodimer with another partner p35 (2). The human IL23-specific p19 subunit is a 189 amino acid polypeptide that contains five cysteine residues and no glycosylation sites. The p19 subunit shows an overall sequence identity of ~40% to the p35 subunit of IL12. The identical p40 subunit of both cytokines binds to the receptor (R) IL12Rβ1, the p35 subunit of IL12 binds to the IL12Rβ2 subunit, and the p19 subunit of IL23 binds to the unique IL23R subunit. The IL23 transmembrane receptor belongs to the class I cytokine receptor family, albeit that it lacks the characteristic membrane-proximal fibronectin type III-like domains. The human IL23 receptor chains are predominantly co-expressed on activated and memory T cells and NK cells, but also at low levels on monocytes, macrophages and dendritic cell (DC) populations. IL23 binds to and signals through the heterodimeric IL12Rβ1/IL23R complex, which is associated with Tyk2 and Jak2, respectively. Upon Jak2-mediated phosphorylation of tyrosine residues located in the intracellular domain of the IL23R subunit, Stat3 molecules are phosphorylated in turn. Phospho-Stat3 proteins homodimerize and translocate into the nucleus, inducing transcription of cytokines such as IL17A, IL17F, IL22, and IFN-γ.

Similar to IL12, IL23 is expressed predominantly by activated DCs and phagocytic cells. IL23 is also produced by antigen-presenting cells and promotes the expansion and survival of a distinct lineage of T cells, Th17 (3). IL17, a proinflammatory cytokine predominantly produced by activated T cells (by Th17 cells), enhances T-cell priming and stimulates fibroblasts, endothelial cells, macrophages, and epithelial cells to produce multiple proinflammatory mediators, including IL1, IL6, TNF-α, NOS2, metalloproteases, and chemokines, resulting in the induction of inflammation (4–6). IL17 expression is increased in patients with a variety of allergic and autoimmune diseases, such as rheumatoid arthritis, multiple sclerosis, inflammatory bowel disease, and asthma, suggesting the contribution of IL17 to the induction and/or development of such diseases. Th17/ThIL17 cells are likely to play critical roles in the development of autoimmunity and allergic reaction, and the IL23/IL17, but not IL12/IFN-γ, axis is critical for the development of autoimmune inflammatory diseases (7).

Our aim was to generate nanobody constructs able to neutralize human interleukin (hIL) 12 and/or hIL23, which could be drug candidates for treatment of Crohn’s disease, rheumatoid arthritis, or psoriasis. In this report, we describe the generation and characterization of nanobodies against p40 and the human (h) IL23-specific p19 subunit. Binding and neutralization characteristics of four selected monovalent nanobodies were determined *via* biochemical and *in vitro* cell assays. As nanobodies have proven to be valuable tools for crystallization purposes in academic contexts (8, 9) as well as in biotechnological or biopharmaceutical contexts (10, 11), crystal structures of hIL23 in complex with three of those four nanobodies were generated. The structure of this quaternary complex helped to understand the high binding efficiency and blocking capacity of the nanobodies. As a result, we were able to rationalize the construction of multivalent nanobodies, whereby the two anti-p19 nanobodies were linked to improve potency and hooked up to an antihuman serum albumin (HSA) nanobody to increase the exposure time (12). The multivalent nanobodies displayed significant enhanced *in vitro* potency in neutralizing IL23 and proved to be very effective in an *in vivo* splenocyte assay performed in mice; hence, they are promising drug candidates.

## Materials and Methods

### IL23 Materials

Human interleukin 23 was purchased from R&D Systems Inc. and from eBioscience. hIL12, mouse (m) IL23, hIL23 receptor (R)-fragment crystallizable region (Fc), and IL12Rβ-Fc chimera were purchased from R&D Systems Inc. Cynomolgus monkey (cyno) IL23 was produced at Ablynx, Ghent/Zwijnaarde, Belgium. hIL23 (eBioscience) and hIL12 (R&D Systems Inc.) were biotinylated using Sulfo-NHS-LC-Biotin (Pierce™).

### Immunization, Library Construction, and Selection of Nanobodies Directed toward hIL23

Two *Lama glama*s were injected with recombinant hIL23, and two llamas received a cocktail of proteins containing a.o. recombinant hIL23 and hIL12. Each animal received seven doses of intramuscular injected antigen at weekly intervals, as described by Roovers et al. (13, 14). Pre-immune and immune sera were collected at day 0, and after 3 and 6 weeks of immunization. The immune response in each animal was monitored by titration of serum samples on coated hIL12 or hIL23.

RNA was prepared from peripheral blood mononuclear cells (PBMCs) isolated from sera and lymph node biopsies. Library constructions were performed as described previously (13, 14), amplifying the variable heavy (V_H_) chain domains of heavy chain antibody (V_HH_) genes and ligating them into the phagemid vector pAX51 in frame with a C-terminal c-myc and hexa-histidine tag for display on phage.

After superinfection of the *Escherichia coli* TG1 library clones with helper phage, the presence of pAX51 allows for the production of phage particles displaying the individual nanobodies as a fusion protein with the pIII protein. Nanobodies recognizing specifically the p19 subunit of hIL23 were retrieved, allowing phages bind to coated hIL23 (5 or 0.5 nM) on microtiter plates. Phages were counter selected three times by binding to wells coated with 5 µg/ml hIL12 to remove p40-binding phages, and further preincubated with 1 µM hIL12 in solution before adding to the hIL23 coated wells. Phages were specifically eluted with trypsin (in the case of 124C4) or with 5 nM of recombinant IL23R (in the case of 37D5). Nanobodies recognizing specifically the p40 subunit of hIL23 and hIL12 were obtained by trypsin elution of phages bound to coated hIL23 (0.1 nM). Subsequently, exponentially growing *E. coli* TG1 cells were infected with the eluted phages, and individual clones were selected, grown in 96-deep well plates (1 ml volume) and induced by the addition of isopropyl-β-d-thiogalactopyranoside (IPTG) for the production of nanobodies. Since the nanobodies are secreted into the periplasmic space, this fraction was then prepared by freeze-thawing of the bacterial pellet in a phosphate-buffered saline (PBS; 100 µl) solution and centrifugation to remove cell fragments.

### ELISA-Binding Screen

1 µg/ml of cytokine (hIL12, hIL23) was immobilized directly on microtiter plates. Free-binding sites were blocked using 4% Marvel in PBS. To this, 5 µl of nanobody containing periplasmic extracts in 100 µl 2% Marvel PBS with Tween 20 were added. Nanobody binding was revealed using a mouse-anti-myc primary antibody, and a horseradish peroxidase-conjugated goat anti-mouse secondary antibody.

### AlphaScreen-Based Receptor-Blocking Assay

To determine the capacity of the nanobodies to inhibit the IL23/IL23R or IL12/IL12Rβ1 interaction, a protein-based competition was used. First, periplasmic extracts were screened for the presence of neutralization capacity at 1 dilution (25-fold), and therefore preincubated with 3 nM biotinylated hIL23 or hIL12. Second, the potency of the neutralizing nanobodies was determined by using serial dilutions of purified p19 blocking (p19+) nanobodies (from 250 nM to 1 pM) or of p40 blocking (p40+) nanobodies (from 250 nM to 1 pM) preincubated with 500 pM biotinylated hIL23 and with 3 nM biotinylated hIL12, respectively. To these mixtures, IL23R, respectively IL12Rβ1 acceptor beads (receptors bound *via* an antihuman Fc monoclonal antibody) and the streptavidin-coated donor beads (Perkin Elmer Inc)—were added, and further incubated for 1 h at room temperature. Fluorescence was measured using the EnVision Multilabel Plate Reader (Perkin Elmer Inc.) using an excitation wavelength of 680 nm and an emission wavelength of 520 nm. Decrease in the AlphaScreen signal indicated that binding of biotinylated hIL23 to the IL23R or hIL12 to the IL12Rβ1 is blocked by the nanobody.

### Surface Plasmon Resonance (SPR)

Surface plasmon resonance was performed using a Biacore T100 instrument. hIL23 was covalently bound to a CM5 sensor chip surface *via* amine coupling using EDC/NHS for activation and HCl for deactivation. Nanobody binding was assessed using periplasmic extracts diluted 1/10 for off-rate determination, and using purified nanobodies at concentrations ranging from 1 to 300 nM for *K*_d_ determination. Each nanobody was injected for 4 min at a flow rate of 45 µl/min to allow binding to chip-bound antigen. Binding buffer without nanobody was then passed over the chip at the same flow rate to allow spontaneous dissociation of bound nanobody. *k*_off_ values were calculated from sensorgrams obtained for the different nanobodies, and *k*_on_ and *K*_d_ values were calculated from sensorgrams for the purified nanobodies. For binning of nanobodies, hIL23 was captured *via* a p40-binding nanobody, which was immobilized on a chip. After binding of one of the lead nanobodies at 500 nM, RU levels were determined to evaluate whether their levels had increased after the flowing over of the other lead nanobody at 500 nM.

### Cloning, Expression, and Purification of Anti-IL23 Nanobodies

Nanobody genes were subcloned into the pAX55 expression vector in frame with a N-terminal *ompA* sequence, and a C-terminal c-myc and hexa-histidine tag (Ablynx; described in WO2008043821). Multivalent nanobody constructs, as outlined in Table 1 (D), were made in the pAX55 vector. They comprise of one or two anti-p19 nanobody building blocks and one building block corresponding to an anti-HSA nanobody building block (ALB1). The individual building blocks were fused by Gly/Ser linkers: 9GS (GGGGSGGGS) or 15GS (GGGGSGGGGSGGGGS). Non-suppressor *E. coli* TG1 cells (Stratagene Corp.) transformed with the appropriate vector were grown at 37°C in Terrific Broth medium supplemented with 100 μg/ml kanamycin and 0.1% glucose for 3 h until optical density (OD_600_) reached ~4. Nanobody expression was induced by the addition of 1 mM IPTG, and growth of the cells was continued for 3–4 h at 37°C. The periplasmic fraction was prepared according to the methods of Skerra and Plückthun (15), and His-tagged nanobodies were purified by immobilized metal affinity chromatography on a 1 ml Ni-NTA column.

**Table 1:** Interactions of the various nanobodies constructs with ILs.

(A) Nanobody types identified at screening				

	p19 binder = p19−	p19 blocker = p19+	p40 binder = p40−	p40 blocker = p40+
Binding to hIL23	+	+	+	+
Binding to hIL12	−	−	+	+
hIL23/IL23R blocking	−	+	−	−
hIL12/IL12Rβ1 blocking	−	−	−	+

**(B) Surface plasmon resonance experiments: *k*_on_, *k*_off_, and *K*_d_ of human p19 and p40 nanobodies binding to hIL23**				

**Nanobody**	**Specificity**	***k*_off_ hIL23 (s^−1^)**	***k*_on_ hIL23 (M^−1^ s^−1^)**	***K*_d_ hIL23 (M)**

37D5	p19+	1.8 × 10^−4^	3.2 × 10^5^	0.6 × 10^−9^
124C4	p19−	3.3 × 10^−4^	1.0 × 10^5^	3.3 × 10^−9^
22E11	p40+	2.3 × 10^−4^	nd	nd
80D10	p40−	9.7 × 10^−5^	5.1 × 10^4^	0.8 × 10^−9^

**(C) IC_50_ values of the antihuman p19 and p40 nanobodies in AlphaScreen**				

**Human p19+ nanobody**		**IC_50_ (pM) hIL23 (confidence range)**		

37D5		110 (80–170)		

**Human p40+ nanobody**		**IC_50_ (pM) hIL12 (confidence range)**		

22E11		1,300 (850–1,950)		

**(D) Splenocyte assay (performed in triplicate)**				
(i) Average IC_50_ values of human p19 and p40 nanobodies using 19 pM hIL23 (±10%) (ii) Comparison of potencies of human p19 formatted, half-life extended nanobodies with 19 pM hIL23 (±10%)				

**Nanobody ID: P23IL**	**Specificity**	**Nanobody construct**		**IC_50_ (pM)**

**(i) Monovalent**				
37D5	p19 blocker	37D5		19
22E11	P40 blocker	22E11		186
**(ii) Multivalent**				
0050	p19 blocker	37D5-**9**GS-Alb1		29.8
0051	p19 blocker + p19 binder	37D5-**9**GS-Alb1-**9**GS-124C4		17
0053	p19 blocker + p19 binder	37D5-**9**GS-Alb1-**15**GS-124C4		3.1
0054	p19 binder + p19 blocker	124C4-**15**GS-Alb1-**9**GS-37D5		4.2
0070	p19 binder + p19 blocker	37D5-**15**GS-Alb1-**15**GS-124C4		3.8
0072	p19 binder + p19 blocker	124C4-**15**GS-Alb1-**15**GS-37D5		3.2
0409	P40 blocker + p40 binder	22E11-**9**GS-Alb1-**15**GS-80D10		0.6

*h, human; IC_50_, half maximal inhibitory concentration; IL, interleukin; nd, not determined; R, receptor; hIL, human interleukin. In D(ii), bold numbers emphasize the length of the GS repeats*.

For crystallization purposes, eluted fractions in 250 mM imidazole were concentrated on an Amicon-Ultra 10 kDa cutoff concentrator prior to being loaded on to a HiLoad 10/30 Superdex75 gel filtration column in Dulbecco’s PBS (dPBS; Invitrogen). Protein concentration of the nanobodies was determined by UV spectrometry from the absorbance at 280 nm, using their calculated extinction coefficient. For the nanobodies that had to be tested in the splenocyte assays, lipopolysaccharide (LPS) removal was performed by ion exchange in flow-through mode or by gel filtration in the presence of octylglucosylpyranoside; remaining LPS levels were determined using a limulus amebocyte lysate assay.

### IC_50_ Determination in a Mouse Splenocyte Assay

Spleens of five C57BL/6 mice were removed, splenocytes were harvested, and a single cell suspension was prepared. Splenocytes were washed three times in RPMI supplemented with 10% fetal bovine serum, 1% pen/strep, 80 µM β-mercaptoethanol, and 1 mM sodium pyruvate. This medium is further referred to as complete RPMI. The splenocyte suspension was treated with 1× erythrocyte lysis buffer (155 mM NH_4_Cl, 10.9 mM KHCO_3_, 1.3 mM EDTA) to remove all residual erythrocytes. After three wash steps in complete RPMI, the cells were filtered over a 100 µM cell strainer and resuspended in complete RPMI containing 20 ng/ml recombinant mouse interleukin (mIL) 2 (R&D Systems Inc.). Cells were seeded at 400,000 cells/well in 96-well flat bottom plates. Serial dilutions of the nanobodies were preincubated with recombinant hIL23 (eBioscience) in culture medium for 30 min at room temperature and then incubated for a further 5 days with the splenocytes, at a final concentration of 19 pM hIL23. Supernatants were collected, and levels of mIL22 measured using ELISA (mIL22 ELISA construction kit, Antigenix America, NY, USA). All tests were done in triplicate.

### Acute *In Vivo* Splenocyte Model

Nanobodies were injected subcutaneously (s.c.) into C57Bl/6 mice (*n* = 4) 24 h before the first of three subsequent intraperitoneal (i.p.) injections of 3 µg hIL23 (at times 0, 7, and 23 h). Test items were administered at a 16-fold, 3.2-fold, or 0.64-fold molar excess to each injection of hIL23. After 31 h, mice were sacrificed and spleens removed. Splenocytes were prepared and mIL22 measured as described above.

### hIL12-Dependent Proliferation of PHA Blasts

PHA blasts were derived from cultured PBMC by stimulation with phytohemagglutinin. They were stimulated for 48 h with 300 pg/ml hIL12. The cells were pulsed with 1 μCi/well ^3^H-thymidine for the last 6 h, and the incorporation of ^3^H-thymidine was determined by scintillation counting in the presence of serial dilutions of nanobodies over a range of 10–10^−5^ µg/ml.

### hIL23/Nanobody Complex Purification and Characterization

1 mg hIL23 in dPBS pH 7.2 was incubated with a slight molar excess of three different purified nanobodies for 1 h on ice. The resulting complex was separated from free nanobody excess by gel filtration chromatography on a Superdex75 column in 10 mM HEPES pH 7.0, 150 mM NaCl, and then concentrated to 7.6 mg/ml on an Amicon-Ultra filter (cutoff 50 kDa; Millipore). Mass spectrometry was performed on a matrix-assisted laser desorption/ionization time of flight (MALDI-TOF) mass spectrometer (Bruker Autoflex) according to standard procedures.

### Crystallization and X-Ray Diffraction

Diffraction-quality crystals of the complex were obtained by sitting-drop vapor diffusion at 277 K after 4–30 days in 16.5% PEG 20 K and 0.1 M MES pH 5.9. Crystals belong to the orthorhombic space group P2_1_2_1_2_1_, with unit cell dimensions: *a* = 102.0 Å, *b* = 134.8 Å, and *c* = 138.4 Å. They contain two complexes per asymmetric unit. Crystals were flash frozen to 100 K using 13% glycerol as the cryoprotectant. Diffraction data were collected under standard cryogenic conditions on beamline ID29, using an ADSC Quantum 4 detector at the ESRF synchrotron (Grenoble, France), processed using MOSFLM (16), and scaled with SCALA (17). The crystal structure of hIL23 in complex with three nanobodies was determined from single-wavelength native diffraction experiments by molecular replacement using PHASER (18). Refinement was performed with BUSTER (19). Figures were constructed using the PyMOL Molecular Graphics System, Version 1.5.0.4 (Schrödinger, LLC). Coordinates were deposited with the Protein Data Bank (PDB) with accession code 4GRW.

## Results

### Generation and Selection of p19- and p40-Binding Nanobodies

To generate V_H_ chain domains of Heavy chain antibodies (V_HH_ or Nanobodies^®^) against the IL23 p19 and p40 subunit, llamas were immunized with recombinant hIL23. Subsequently, phage nanobody libraries were generated from PBMCs and lymph node biopsies (LNs) collected from these animals. This resulted in four libraries with sizes between 2 × 10^7^ and 4 × 10^7^, and a percentage of insert containing clones ranging from 91 to 100%. After selection, nanobodies present in the periplasmic fractions of isolated clones were screened for their binding specificity to hIL12 versus hIL23 in an ELISA binding assay. Nanobodies binding to p40 were identified and recognized both hIL12 and hIL23, wheras nanobodies binding to p19 specifically recognized hIL23 only.

Periplasmic extracts were then analyzed to determine the ability of the nanobodies herein to inhibit the hIL23-hIL23R or the hIL12-hIL12Rβ1 interaction *via* an Amplified Luminescent Proximity Homogeneous (AlphaScreen) Assay for protein–protein interaction detection. Nanobodies decreasing the signal in this assay inhibit the hIL23/hIL23R (designated p19+) or the hIL12/hIL12Rβ1 interaction (designated p40+).

Different nanobody types were identified in the binding and neutralization screening assays (Table 1, A). A selection of p19 binding (p19−), p19 blocking (p19+), p40 binding (p40−), and p40 blocking (p40+) nanobodies were DNA sequenced. Twenty families of anti-p19 nanobody sequences were identified, with 7 families containing p19+ and 13 containing p19− nanobodies; and 24 families of anti-p40 nanobody sequences, with 12 families containing p40+ and 12 containing p40− nanobodies. The off-rates of the unique family members were determined using SPR. The family members with the slowest off-rates on hIL23 or hIL12 were recloned and purified for further characterization.

### Affinity Determination of Anti-p19 and Anti-p40 Nanobodies

Binding kinetics of the purified human p19+ nanobody 37D5, the p19− nanobody 124C4, the p40+ nanobody 22E11, and the p40− nanobody 80D10 were determined using SPR. From the sensorgrams obtained, *k*_on_, *k*_off_, and *K*_d_ were calculated where possible (Table 1, B). The three compounds were found to display excellent off-rates for hIL23, with nanobody 37D5 having the best affinity (*K*_d_ 0.57 nM). Species cross-reactivity profiles of the monovalent nanobodies were also studied using SPR. All four nanobodies bound to cynomolgus monkey (cyno) IL23 with similar off-rates, and only nanobody 124C4 was able to cross-react to mouse (m) IL23. Knowledge of species cross-reactivity is important in light of future animal efficacy and pharmacokinetic/pharmacodynamic studies.

### *In Vitro* Evaluation of the Inhibiting Capacity of Anti-p19 and Anti-p40 Nanobodies

The potency of the nanobodies to inhibit the IL23/IL23R or IL12/IL12Rβ1 interaction was determined either in a protein-based competition assay using AlphaScreen or in cell-based potency assays.

For the AlphaScreen, preincubation of a serial dilution of the p19+ nanobody 37D5 with biotinylated hIL23 reduced fluorescence intensity at 520 nm, demonstrating that this nanobody can effectively inhibit hIL23 binding to IL23R in a dose-dependent manner with a half maximal inhibitory concentration (IC_50_) of 110 pM. Preincubation of a serial dilution of the p40+ nanobody 22E11 with biotinylated hIL12 also reduced the fluorescence intensity, demonstrating that this nanobody can effectively inhibit hIL12 binding to IL12Rβ1 with an IC_50_ of 1,300 pM (Table 1, C).

Inhibition of hIL23-mediated signaling by the nanobodies was investigated using a mouse splenocyte assay. This cell-based potency assay is based on the ability of hIL23 to stimulate mIL22 secretion from mouse spleen cells (20). The p19+ nanobody 37D5 appears to be very potent reaching an average IC50 value of 19 pM when using 19 pM hIL23 for stimulation, whereas the p40+ nanobody 22E11 shows an IC_50_ of 186 pM (Table 1, D, i).

### Anti-p19 Nanobodies 37D5 and 124C4 Recognize Different Epitopes

As shown above, nanobody 37D5 shows good neutralizing activity toward hIL23. In theory, it would be possible to further improve the hIL23 neutralization by linking nanobody 37D5 to a p19-binding non-blocking nanobody as avid interaction of such a multivalent construct with the cytokine is expected. To do so, both nanobodies need to bind to different epitopes.

An SPR experiment was conducted where hIL23 was captured *via* a p40-binding nanobody (80D10), which was immobilized on a chip. After binding the p19 blocker (nanobody 37D5), it was assessed as to whether a second nanobody could bind simultaneously to hIL23 p19 subunit. The interaction studies show that nanobody 37D5 can bind concurrently with the p19− nanobody 124C4, since RU levels double upon binding of the second nanobody (Figure S1 in Supplementary Material, green trace). For other p19− nanobodies tested, this was not the case (Figure S1 in Supplementary Material, non-green traces). Hence, nanobody 37D5 and nanobody 124C4 could be combined in a multivalent construct and used simultaneously for crystallization in complex with hIL23.

### Structure of hIL23 Bound Simultaneously to Nanobodies 37D5, 124C4, and 22E11

The high affinity of the monovalent nanobodies and the existence of different epitopes for the p19+ nanobody 37D5, the p19− nanobody 124C4, and the p40+ nanobody 22E11 encouraged us to crystallize recombinant hIL23 in complex with these three nanobodies. hIL23 was mixed with an excess of the nanobodies, and the complex was purified by gel filtration. Crystals were readily obtained, and the structure was solved by molecular replacement using the hIL23 structure [in complex with the Fab 7G10 (21)] and V_HH_ structures (22, 23) as search models. The crystals contain two complexes in the asymmetric unit and the structure was refined to *R*/*R*_free_ values of 18.3%/21.8%, respectively (Table S1 in Supplementary Material).

**Figure 1:**
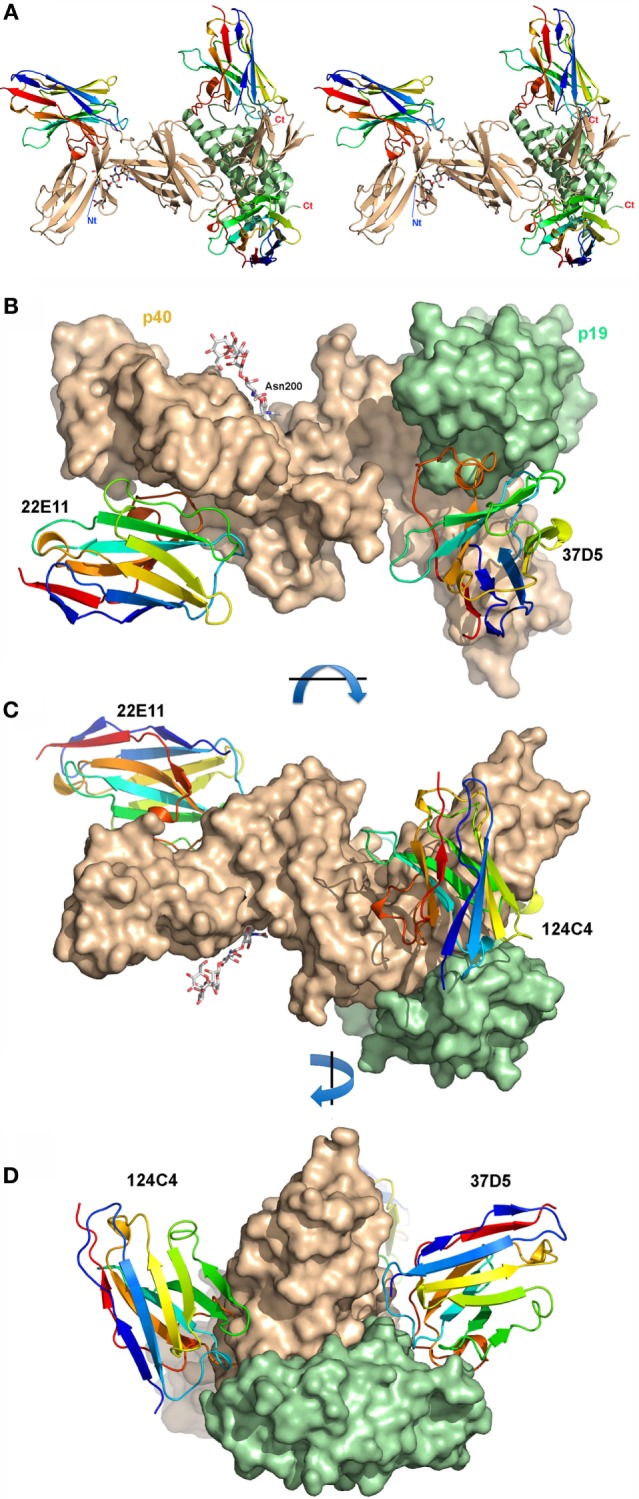
Human interleukin (hIL) 23 in complex with three nanobodies. **(A)** Stereo view of hIL23 in complex with three nanobodies. p40 is shown in brown, p19 green, and the 37D5, 124C4, and 22E11 nanobodies are in rainbow colors. **(B)** Surface representation of hIL23, with the three nanobodies in ribbon representation. The Asn 200 branched sugar is represented by sticks. **(C)** 90° rotation around a horizontal axis [relative to panel **(B)**]. **(D)** 90° rotation around a vertical axis [relative to panel **(C)**].

The overall structure of hIL23 with its three bound nanobodies is depicted in Figure 1. hIL23 is formed by two monomers, p19 and p40, linked by a disulfide bridge between Cys 54 (p19) and Cys177 (p40). The monomer p19 is formed by a four antiparallel helix bundle (21, 24, 25). The p40 protein is formed of three domains, each composed of a 7 β-stranded β-sandwich (2, 21, 24, 25). The interaction surface between p19 and p40 is ~900 Å^2^ and hence quite large. The p19 monomer inserts its fourth helix between the two first domains of p40. The interface is completed by a p19 loop joining helices H1 and H2. The structure of hIL23 in our complex is close to the structures published previously, with root mean square deviation (rmsd) values of 0.7–1.2 Å, mainly due to slightly different orientations of the three p40 domains. The rmsd values observed between the same domains in different structures are indeed much lower (0.5–0.9 Å). Noteworthy, two saccharidic chains of the complex type are observed at Asn 200 of the two p40 chains, with the five core sugars well defined (GlcNAc_2_-Man_3_) (Figure S2 in Supplementary Material). Superposition of the two independent complexes of the asymmetric unit indicates that the two complexes are quite similar (rmsd < 1.0 Å) for p40, nanobody 124C4, and nanobody 22E11 (Figure S3 in Supplementary Material).

**Figure 2:**
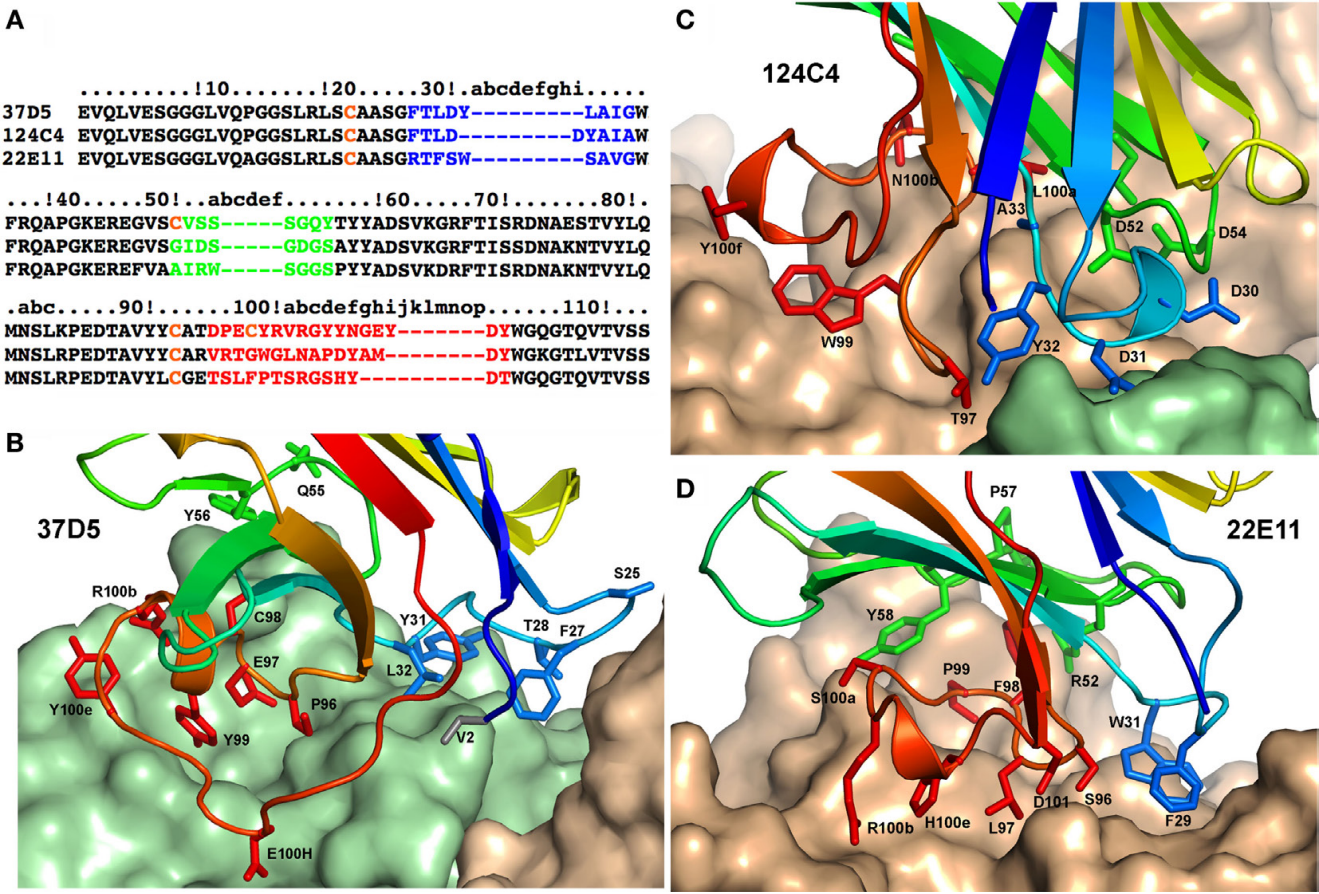
Contacts of human interleukin (hIL) 23 in complex with three nanobodies. **(A)** Sequence alignment of the nanobodies 37D5, 124C4, and 22E11. Complementarity-determining region (CDR) 1 is shown in blue, CDR2 is green, CDR3 is red, and cysteines are shown in orange. **(B)** N-terminal, CDR, and framework 3 residues of nanobody 37D5 that interact with hIL23. **(C)** CDR residues of nanobody 124C4 that interact with hIL23. **(D)** Residues of nanobody 22E11 that interact with p40. In **(B)**–**(D)** p40 is shown in brown, p19 is green, and the 37D5, 124C4, and 22E11 nanobodies are in rainbow colors. CDR1 is shown in green, CDR2 is blue, and CDR3 is red.

Totally unexpected from the interaction studies in solution, the structure reveals that the p19+ 37D5 nanobody and the p19− nanobody 124C4 interact with both subunits of hIL23, p19 and p40 (Figures 1 and 2; Tables S2 and S3 and Figures S4 and S5 in Supplementary Material). As frequently observed with nanobodies (8), nanobody 37D5 and nanobody 124C4 bind to concave surfaces and insert their complementarity-determining regions (CDRs) in the crevices formed at the junction between p19 and p40. In contrast and as expected, the p40+ 22E11 nanobody interacts only with p40 and binds a flat surface of the cytokine’s first p40 domain, remote from the two other nanobodies that bind the third p40 domain.

### Interaction of the Three Nanobodies with hIL23 and Description of a Novel Neutralizing Epitope

The three nanobodies interact with hIL23 with high affinity, which correlates with very large buried surface areas: ~1,110 Å^2^ for nanobody 37D5, ~830 Å^2^ for nanobody 124C4, and ~780 Å^2^ for nanobody 22E11 (Table S2 in Supplementary Material). Nanobody 37D5 was initially assigned as a p19 binder and has, indeed, a larger surface of interaction with p19 (850 Å^2^) than with p40 (260 Å^2^) (Table S2 in Supplementary Material). It binds to the p19 helix bundle with its three CDRs and a few residues from the framework. CDR3, however, exhibits the most extended interaction with seven residues as compared to three residues for CDR1 and CDR2 (Table S3A in Supplementary Material; Figure 2B). The interaction with p40 occurs through three residues (Table S3 in Supplementary Material), with Phe 27 inserted deeply between both monomers. The 124C4 nanobody, which was initially assigned as a p19 binder, exhibits a binding pattern opposite to the one of 37D5, with a larger surface of interaction with p40 (625 Å^2^) than for p19 (200 Å^2^). Interaction with p40 involves four residues of CDR2 and seven residues of CDR3 (Table S3B in Supplementary Material; Figure 2C). Interaction with p19 involves four residues of CDR1 and one residue (Thr97) from CDR3. Finally, nanobody 22E11 interacts exclusively with p40. The interface involves a small number of contacts with CDR1 and CDR2 (two and four residues, respectively) and a large number of contacts (eight residues) from CDR3 (Table S3C in Supplementary Material; Figure 2D).

### Formatting of the Anti-p19 Nanobodies Results in Very Potent hIL23-Neutralizing Molecules

**Figure 3:**
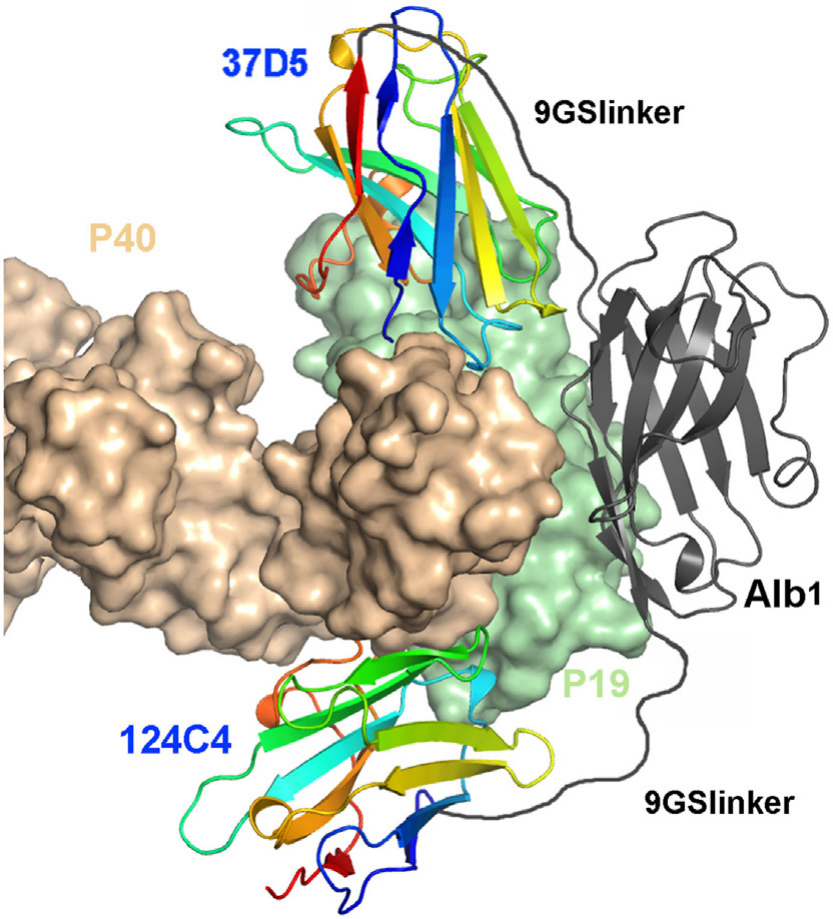
Model of a multivalent construct combining the nanobodies 124C4 and 37D5 with the antihuman serum albumin nanobody Alb1 flanked by two 9GS flexible linkers. This 9GS-Alb1-9GS construct is able to bridge the distance between the C-terminus of 124C4 and the N-terminus of 37D5 when bound to human interleukin 23.

We generated multivalent constructs, whereby we assembled the p19-neutralizing nanobody 37D5 with the non-neutralizing nanobody 124C4. To prolong the half-life of the molecules for use as therapeutic agents in inflammatory diseases, a nanobody that binds serum albumin (Alb1) was included in these constructs. The human-mouse serum albumin cross-reactive nanobody Alb1 was positioned between the two anti-p19 nanobody building blocks (Figure 3). The length of the linkers fusing Alb1 to the anti-p19 nanobody building blocks was varied, based on predictions using the crystal structures. In addition, the nanobody order in the constructs was varied, as N- or C-terminal positioning of a certain building block can influence its binding affinity. Next to that, a half-life extended (HLE) version of nanobody 37D5 on its own (37D5-9GS-Alb1, Table 1, D, ii) was generated, since nanobody 37D5 is already quite potent in a monovalent format. The potency of the different constructs was determined in the mouse splenocyte assay using hIL23 as described above. The potency of 37D5-9GS-Alb1 is slightly decreased; however, potencies of the trivalent nanobodies are better (Table 1, D, ii). When placing nanobody 37D5 at the N-terminal position, the neutralizing potency is approximately fivefold better with a 9 + 15GS linker compared to a 9 + 9GS linker (Table 1, D, ii). When combining 9 + 15GS or 15 + 15GS, the order of the building blocks has little influence on the potency to neutralize hIL23.

### HLE Multivalent Anti-p19 Nanobodies Show Improved Efficacy *In Vivo*

**Figure 4:**
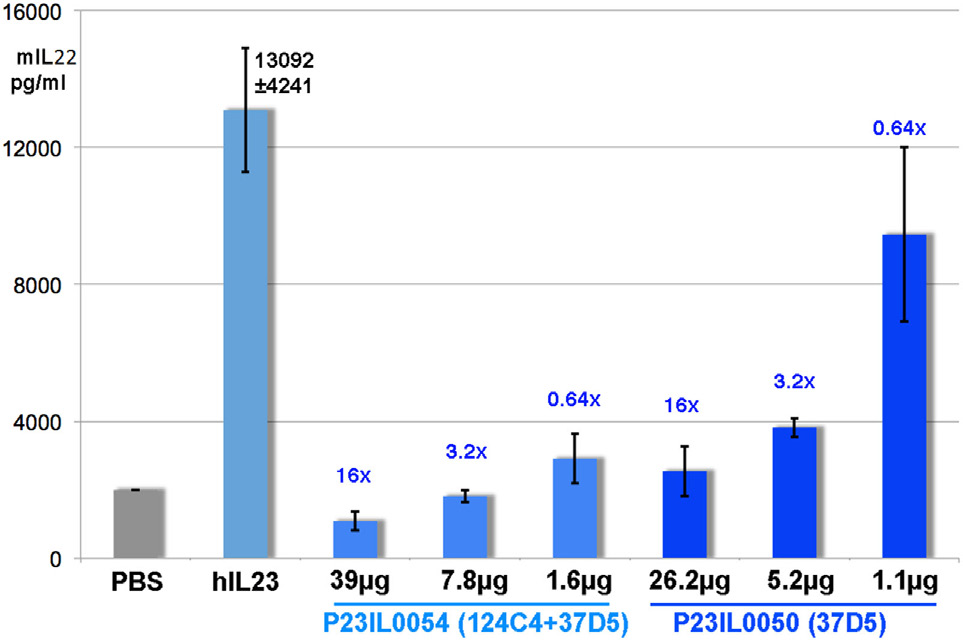
Inhibition of human interleukin (hIL) 23 induced mouse interleukin (mIL) 22 synthesis upon administration of P23IL0050 or P23IL0054. The *y*-axis indicates the average mIL22 synthesis in picograms per milliliter. The *x*-axis depicts the different test groups (two nanobodies, each at three different microgram doses). Numbers in blue positioned above the bars give the molar excess ratio of nanobody administered over the 3 µg hIL23 injected. The number above the hIL23 bar is the mean mIL22 synthesis for the control group receiving hIL23 only (pg/ml ± SD).

An acute *in vivo* mouse splenocyte model assay demonstrated the efficacy of the formatted nanobodies P23IL0050 (37D5-9GS-Alb1) and P23IL0054 (124C4-15GS-Alb1-9GS-37D5). Nanobodies were injected subcutaneously (s.c.) 24 h before the first of three subsequent intraperitoneal (i.p.) injections of 3 µg hIL23 at times 0, 7, and 23 h occurred. Nanobodies were administered at three different doses, at a 16-fold, 3.2-fold, or 0.64-fold molar excess to each injection of hIL23. P23IL0050 and P23IL0054 were capable of neutralizing hIL23 *in vivo*, measured as significant and complete blocking of mIL22 synthesis at the two highest doses tested (Figure 4). Administration of the lowest dose of the monovalent nanobody construct P23IL0050 (0.64-fold excess) blocked hIL23-induced mIL22 production only partially, whereas multivalent construct P23IL0054 still shows significant blocking demonstrating the power of combining different anti-p19 nanobodies in one construct (Figure 4).

## Discussion

### Structural Analysis of Monovalent Nanobodies

**Figure 5:**
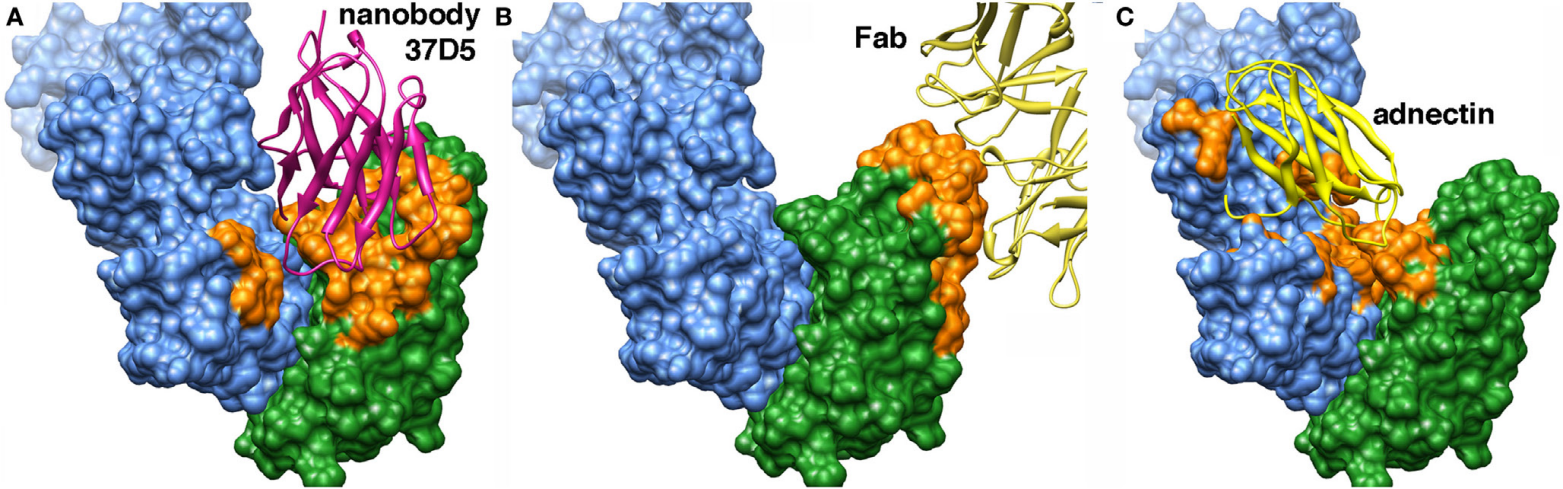
Comparison of three complexes of human interleukin 23 with ligands. The p40 subunit is shown in blue, and p19 is green. The surface of interaction is colored orange. **(A)** Complex with the 37D5 nanobody (shown in pink). **(B)** Complex with the neutralizing Fab 7G10 (partial view; shown in yellow). **(C)** Complex with adnectin (shown in yellow).

We report here the generation and characterization of high-affinity nanobodies derived from llama heavy chain antibodies raised against hIL23. Two of the nanobodies studied revealed a high efficacy in neutralizing hIL23 in *in vitro* bioassays using mouse splenocytes. The IC_50_ values ranged from ~20 to ~200 pM. To better understand the mode of action of those neutralizing nanobodies, the crystal structure of hIL23 in complex with the nanobodies 37D5 (p19+), 22E11 (p40+), and 124C4 (p19−) was generated. Comparison of our structure of hIL23, with the already known structures, i.e., hIL23 alone [3DUH (24)], hIL23 in complex with the Fab fragment (7G10) of a neutralizing antibody [3D85 (21)], and hIL23 in complex with adnectin [3QWQ (25)], shows that all structures are very similar (Figure 5). The rmsd values of p19 are between 1.0 and 1.2 Å, whereas those of p40 range are between 1.0 and 1.9 Å (Table S4 in Supplementary Material).

We compared the binding mode of three p19-neutralizing molecules: nanobody 37D5, Fab 7G10, and adnectin to hIL23 (Figure 5; Figure S5 in Supplementary Material). Fab 7G10 binds exclusively to p19, and the surface covered by the V_H_ and variable light (V_L_) chains is convex, as expected from this type of binder. In contrast, both the nanobody 37D5 and adnectin bind concomitantly p19 and p40. They recognize a concave surface, a result often observed for nanobodies (8). When looking at the interaction surface areas [all measured by the same server, PISA (26)], adnectin is by far covering the largest surface area (1,566 Å^2^), followed by nanobody 37D5 (870 Å^2^), and Fab 7G10 (778 Å^2^) (Table S5 in Supplementary Material). As those molecules are neutralizing, and hence competing with IL23R interaction, they probably overlap with the IL23R epitope. To the best of our knowledge, no structure of an IL23-IL23R complex is known to date, but some suggestions on possible IL23R epitopes have been described (21, 24).

To understand the fact that nanobody 124C4 and nanobody 37D5 were initially expected to be solely p19 binders, but bound both p19 and p40, we superimposed our hIL23 structure on that of hIL12 [pdb 1f45 (2)]. IL12 has the p40 subunit in common with IL23 and forms a heterodimer with a p35 subunit, instead of p19 in IL23. There is a good overlap between the two p40 subunits and also the secondary structure elements of p19 overlap well with p35 (backbone rmsd = 1.6 Å). However, since p35 is larger than p19, some exposed regions of p40 in hIL23 are buried in hIL12. Indeed, due to the larger size of p35 versus p19, the binding mode of nanobody 124C4 to IL23 is not compatible with the p35 position and surface in IL12 (Figure S4 in Supplementary Material).

The 22E11 nanobody binds the N-terminal domain of the p40 monomer. Interestingly, its binding area overlaps with that of the Fab of Ustekinumab (Figure S6 in Supplementary Material; PDB: 3HMX) (27, 28). The antibody Ustekinumab targets p40, neutralizes both IL12 and IL23, and is marketed for treatment of psoriasis. Consistent with the overlap in epitopes, the anti-p40 blocking nanobody 22E11 is indeed able to neutralize hIL12 in addition to hIL23, as it could block hIL12-dependent proliferation of PBMCs stimulated with phytohemagglutinin (so called PHA blasts) with an IC_50_ of 95 pM when 4 pM hIL12 was used for stimulation (data not shown). Although the 22E11 nanobody is not as potent as Ustekinumab in neutralizing hIL23, it can be presumed that fusing nanobody 22E11 with an anti-p40 nanobody, recognizing an epitope covering the Ustekinumab p40-binding region not overlapping with 22E11, will significantly improve potency. This hypothesis is supported by a multivalent construct consisting of nanobody 22E11 and a p40 nanobody (nanobody 80D10), recognizing a non-neutralizing epitope. This construct (22E11-9GS-Alb1-15GS-80D10) improves the potency of the monovalent 22E11 by 300-fold (IC_50_ of 0.6 pM in the splenocyte assay, Table 1, D, ii).

However, it is preferred to target p19, and hence being specific for IL23, as targeting IL12 could potentially lead to infection-related side effects.

### Use of Multivalent Nanobodies to Improve Efficacy

Several multivalent constructs were engineered by flanking Alb1 by a linker on each side, followed by 37D5 or 124C4 on either side, in an attempt to improve the potency/efficacy of the 37D5 nanobody. As control 37D5, fused to the anti-HSA nanobody Alb1, was used. Analysis of the X-ray structure of hIL23 in complex with nanobody 37D5 and nanobody 124C4 was used to predict the peptide linker lengths needed to combine these two nanobodies in a multivalent construct, enabling simultaneous binding of both nanobodies to hIL23. The direct distance between the C-terminus of nanobody 37D5 and the N-terminus of nanobody 124C4 is ~85 Å, indicating that at least a 25GS linker is needed in a bivalent construct. As the Alb1 nanobody was introduced between the anti-p19 nanobodies, it served as part of the linker and was flanked by two 9-mer linkers or a 9- and 15GS linker. Based on protein modeling we concluded that although a (-9GS-Alb1-9GS-) linker might be sufficient to permit binding of both nanobodies, a (-9GS-Alb1-15GS-) provides more flexibility to the construct allowing the Alb1 nanobody to accommodate better with respect to the p19 and p40 units. This flexibility seemed to be important since experimentally, when Alb1 was flanked by a 9- and 15GS linker, the IC_50_ value in the splenocyte assay for hIL23 was significantly better than that obtained with two 9GS linkers. Furthermore, when having 9 + 15GS or 15 + 15GS, the potency became independent of nanobody orientation, with values between 3.2 and 3.8 pM (Table 1, D, ii).

*In vivo*, the multivalent construct P23IL0054 (124C4-15GS-Alb1-9GS-37D5) was more efficient in neutralization of administrated hIL23 to mice (Figure 4), than the monovalent p19 blocker 37D5 fused to Alb1, confirming the power of formatting nanobodies. In addition, P23IL0054 is presumably specific for IL23, unless a new heterodimeric cytokine using p19 in conjunction with another subunit emerges.

In conclusion, the multivalent approach designed and reported here has two exquisite beneficial effects. First, formatting leads to avid binding to hIL23, leading to a 10-fold increase in potency, compared to the monovalent p19 blocker. Second and as important, by acting on two different epitopes, chances to retain the specific binding to the heterodimeric hIL23 are increased, avoiding undesired cross-reactivity with other cytokines (known or still unknown) sharing one of its components. The formatting power of the nanobody platform, together with a long *in vivo* residence time and low immunogenicity profile, raises the opportunity for the best of these nanobody constructs to become excellent drug candidates to treat inflammatory diseases.

## Data Deposition

Coordinates were deposited with the Protein Data Bank with accession code 4GRW.

## Ethics Statement

Animal work was conducted according to the guidelines set out in European Economic Community council directive 86/609/EEC, with authorization from the prefecture of Doubs and the Department of Veterinary Services.

## Author Contributions

HH, MS, HR, and CC designed the experiments. AD, SS, and CC purified, crystallized, and solved the X-ray structures. CB analyzed the X-ray structures and identified molecular interactions. GD, ChB and HR were responsible for nanobody generation and characterization. All the authors contributed to the analysis of the results and writing of this paper.

## Conflict of Interest Statement

This research has been funded by Ablynx, with the exception of research by AD, SS, and CC at the Architecture et Fonction des Macromolécules Biologiques (France), whose work has been funded in part by Ablynx and the Centre National de la Recherche Scientifique (France), and the Aix-Marseille Université (France).

## Abbreviations

V_HH_, variable heavy chain domain of llama heavy chain antibody; CDR, complementarity-determining region; Nb, nanobody; SPR, surface plasmon resonance; *K*_d_; equilibrium dissociation constant; *k*_on_, association rate constant; *k*_off_, rate constant; rmsd, root mean square deviation; GS linker, glycine–serine linker.
