# Supplementary material for: Neutralization of Human Interleukin 23 by Multivalent Nanobodies Explained by the Structure of Cytokine–Nanobody Complex
Source: Front Immunol. 2017 Aug 21;8:884. doi: 10.3389/fimmu.2017.00884 (PMC5566574; doi:10.3389/fimmu.2017.00884)
Supplement: Supplementary file 1 [file table_1.pdf]

Supplementary Material

Neutralization of hIL23 by multivalent Nanobodies explained by the structure of cytokine-Nanobody complex

Aline Desmyter[#1,2], Silvia Spinelli[#1,2], Carlo Boutton[3], Michael Saunders[3†], Hans de Haard[3†], Geertrui Denecker[3§], Maarten Van Roy[3], Christian Cambillau[1,2*] and Heidi Rommelaere[3*]

* Correspondence:
Dr Heidi Rommelaere: Heidi.Rommelaere@ablynx.be
Dr Christian Cambillau: cambillau@afmb.univ-mrs.fr

Supplementary table

Supplementary table1. Data collection and refinement statistics.

**Data Collection**	
Beamline	ID29-ESRF
Resolution limits (Å)	45.00–2.54 (2.61–254)[a]
Rmerge[a] (%)	8.2 (60.0)[a]
Observations (n)	257073 (20269)[a]
Unique reflections (n)	63758 (4921)[a]
Mean ((I) / sd(I))	13.5 (2.65)[a]
Completeness (%)	99.8 (100)[a]
Multiplicity	4.0 (4.1)[a]
**Refinement**	
Resolution (Å)	43.83–2.55 (2.62–2.55)[a]
Reflections (n)	63328 (4494)[a]
Atoms protein/sugar/water	12294/122/738
Test set reflections (n)	1940
R_{work} / R^{freea} (%)	18.4 / 21.8 (22.3 / 25.0)[a]
r.m.s.d.bonds(Å)/angles (°)	0.010 / 1.21
B-average / B-Wilson	50.5 / 55.4
Ramachandran, Preferred, allowed, outliers (%)	96.0 / 3.0 / 1.0

[a] highest resolution bin shown in parenthesis.

Supplementary table 2. Overall interactions of individual Nanobodies with hIL23. BSA, buried surface area; hIL23, human interleukin 23; PDB, protein data bank.

	**Monomers in PDB** *Complex1*	**BSA ($Å^2$)**	**DG-calc (kcal/mole)**	**Monomers in PDB** *Complex2*	**BSA ($Å^2$)**	**DG-calc (kcal/mole)**	**Kd (nM)**
p19-p40	*A-B*	*913*	*-11.1*	*C-D*	*905*	*-9.5*	
37D5-p19	J-A	860	-10.7	F-C	840	-10.1	
37D5-p40	J-B	273	-0.7	F-D	245	-0.6	
**37D5**	**J**	**1133**	**-11.7**	**F**	**1085**	**-10.6**	**0.57**
124C4-p40	G-A	618	-1.6	E-D	630	-1.6	
124C4-p19	G-B	176	-0.3	E-C	240	-0.4	
**124C4**	**G**	**794**	**-1.9**	**E**	**870**	**-2.0**	**3.3**
**22E11-p40**	**H-B**	**755**	**-5.0**	I-D	**800**	**-3.6**	**nd**

Supplementary table 3. Residues (Kabat numbered) from the three Nanobodies in contact with IL23 p19 or p40.

**A**	***37D5 residues in contact with p19***	***37D5 residues in contact with p40***
N-terminus	V2	E1
CDR-1	T28, Y31, L32	S25, G26, F27
CDR-2	S52, Q55, Y56	
Framework 3	E75, S76	
CDR-3	P96, E97, C98, Y99, R100b, Y100e, E100h	
**B**	***124C4 residues in contact with p19***	***124C4 residues in contact with p40***
CDR-1	D30, D31, Y32, A33	
CDR-2		D52, D54, G55, S56
CDR-3	T97	T97, G98, W99, G100, L100a, N100b, Y100f
**C**		***22E11 residues in contact with p40***
CDR-1		F29, W31
CDR-2		R52, S56, P57, Y58
CDR-3		S96, L97, F98, P99, S100a, R100b, H100e, D101

Supplementary table 4. Structural similarities as reported by the DALI server (rmsd in Å; amino-acids in the alignment/ total amino-acids).

	3D85, IL23	3D87, IL23	3QWR; IL23	3DUH, IL12
p19 / beta	1.2; 126/133	1.0; 128/159	1.1; 122/141	0.7; 118/137
p40	1.7; 288/290	1.9; 288/296	1.0; 289/295	1.3; 288/294

Supplementary table 5. Buried surface areas (BSA) in $Å^2$ at the interfaces of hIL23 and Nanobodies (this study) or other binders.

BSA value in $Å^2$ by PISA [REF]	p19	p40	Sum
hIL23 – 37D5	850	260	1110
hIL23-124C4	240	630	870
hIL23-22E11		800	800
hIL23 - Fab	778 (332 / 446)		778
hIL23 - Adnectin	629	937	1566
