# Supplementary material for: Neutralization of Human Interleukin 23 by Multivalent Nanobodies Explained by the Structure of Cytokine–Nanobody Complex
Source: Front Immunol. 2017 Aug 21;8:884. doi: 10.3389/fimmu.2017.00884 (PMC5566574; doi:10.3389/fimmu.2017.00884)
Supplement: Supplementary file 2 [file image_1.pdf]

Supplementary Material

Neutralization of hIL23 by multivalent Nanobodies explained by the structure of cytokine-Nanobody complex

Aline Desmyter[#1,2], Silvia Spinelli[#1,2], Carlo Boutton[3], Michael Saunders[3†], Hans de Haard[3†], Geertrui Denecker[3§], Maarten Van Roy[3], Christian Cambillau[1,2]* and Heidi Rommelaere[3]*

* Correspondence:
Dr Heidi Rommelaere: Heidi.Rommelaere@ablynx.be
Dr Christian Cambillau: cambillau@afmb.univ-mrs.fr

Supplementary Figures

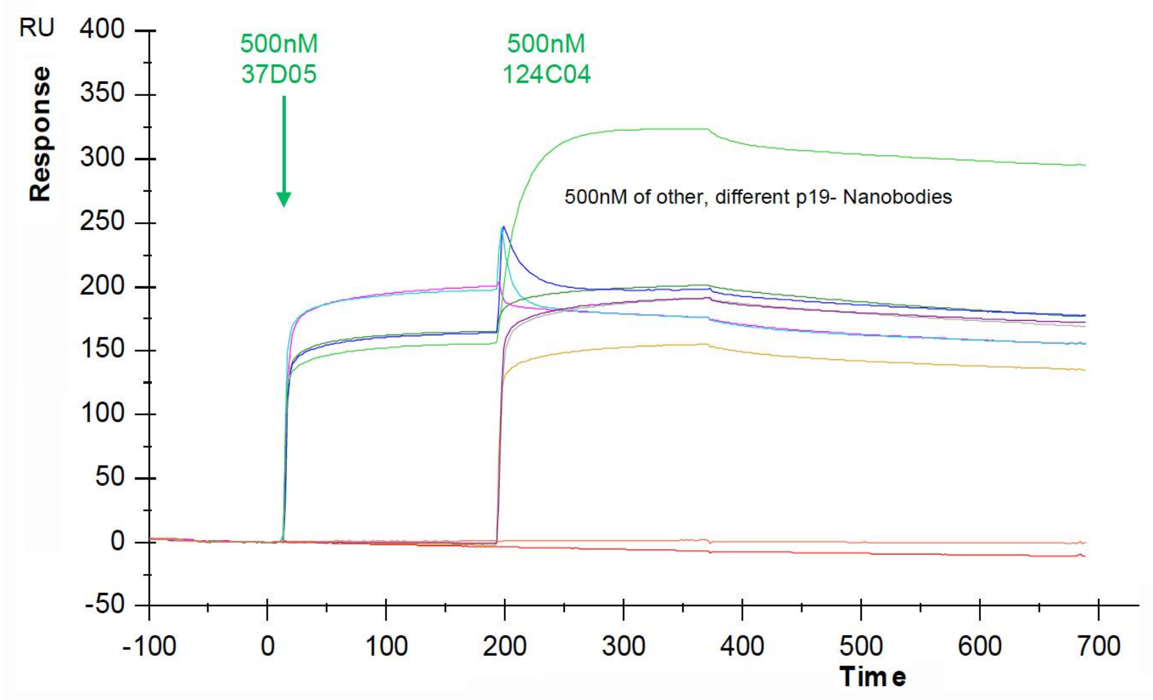

Supplementary figure 1. BiaCore Sensorgram of the epitope binding experiment with Nanobodies 37D5 and 124C4.

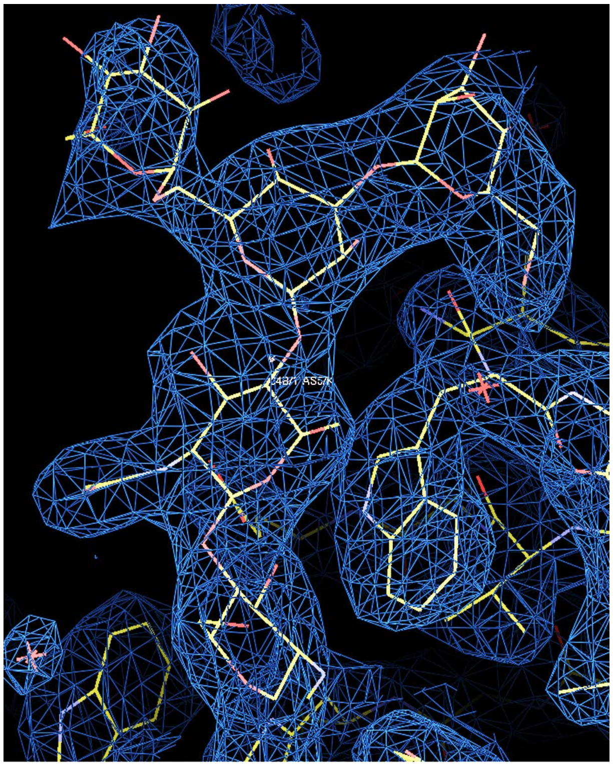

Supplementary figure 2. Electron density map of the biantennary saccharide attached to Asn200. The map is contoured at 1 sigma level.

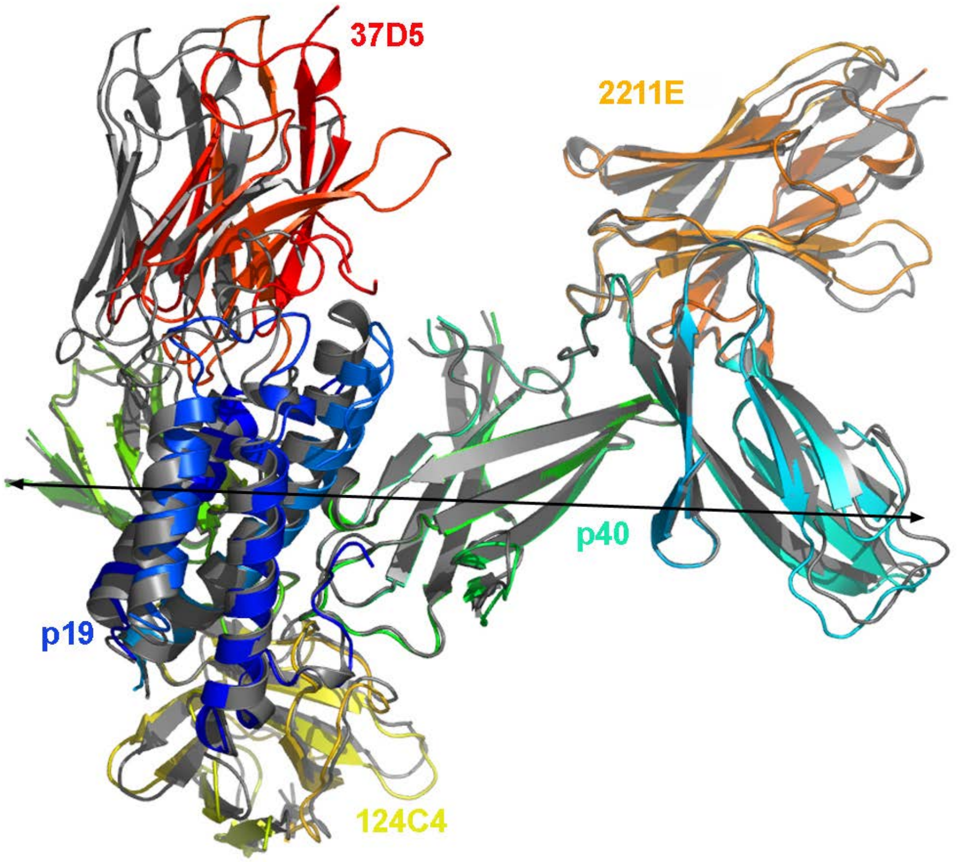

Supplementary figure 3. Comparison of the two quaternary complexes in the asymmetric unit. One complex in the asymmetric unit is coloured grey, and the other is shown in rainbow colouring: p19, dark blue; p40, light blue to green; Nb 3D75, red; Nb 124C4, yellow; and Nb 22E11, orange.

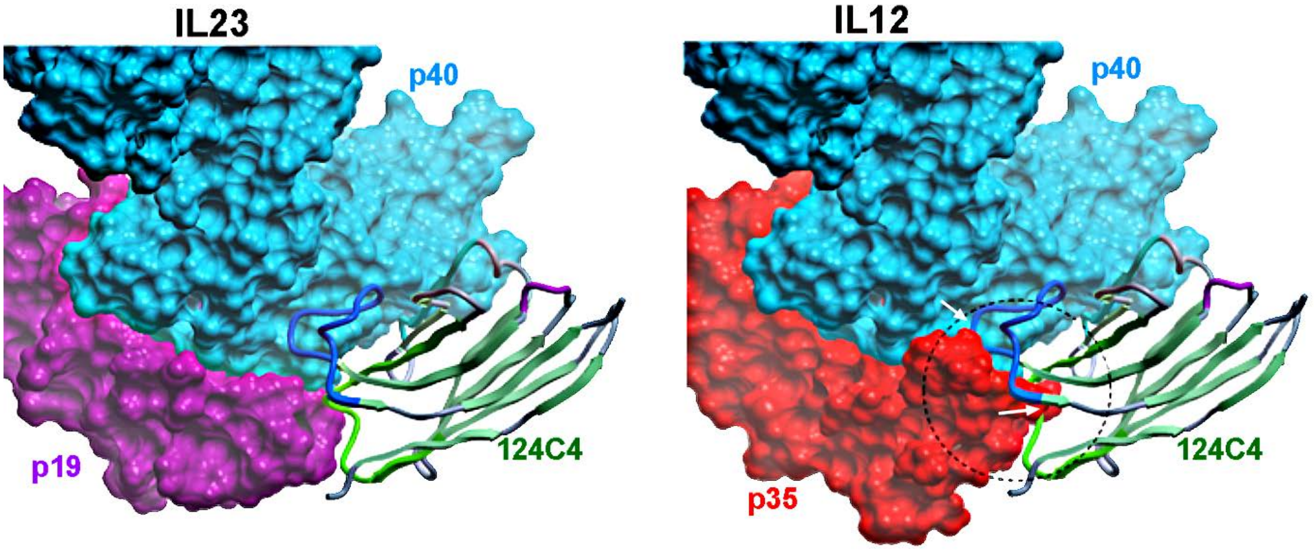

Supplementary figure 4. Comparison Nb 124C4 binding to IL12 with Nb 124C4 binding to IL23. IL12, with p40 shown in blue, p19 in purple, and p35 shown in red. The dotted circle and the arrows indicate clashes that would prevent binding of Nb 124C4 to IL12.

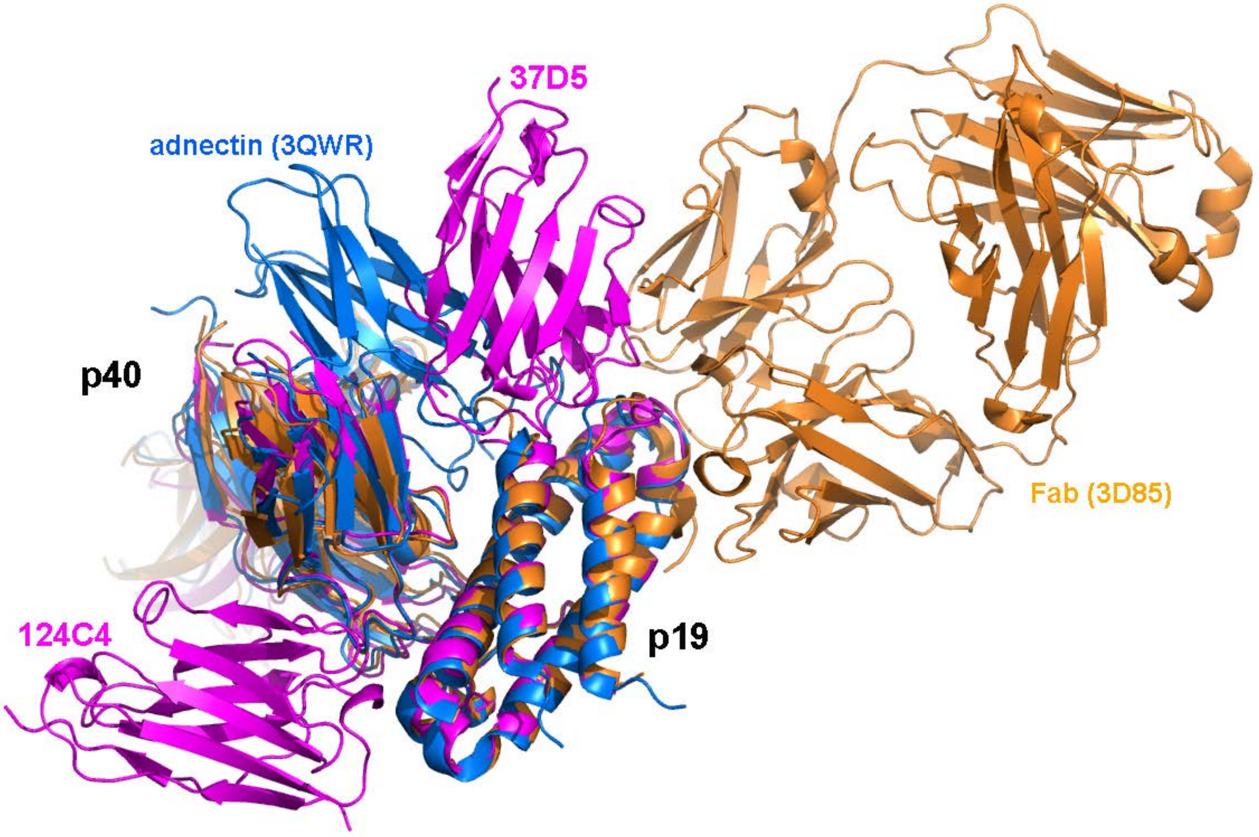

Supplementary figure 5. Comparison of the three complexes of hIL23 with ligands. The complex with three Nanobodies is shown in pink, the Fab complex in brown, and the adnectin complex is shown in blue.

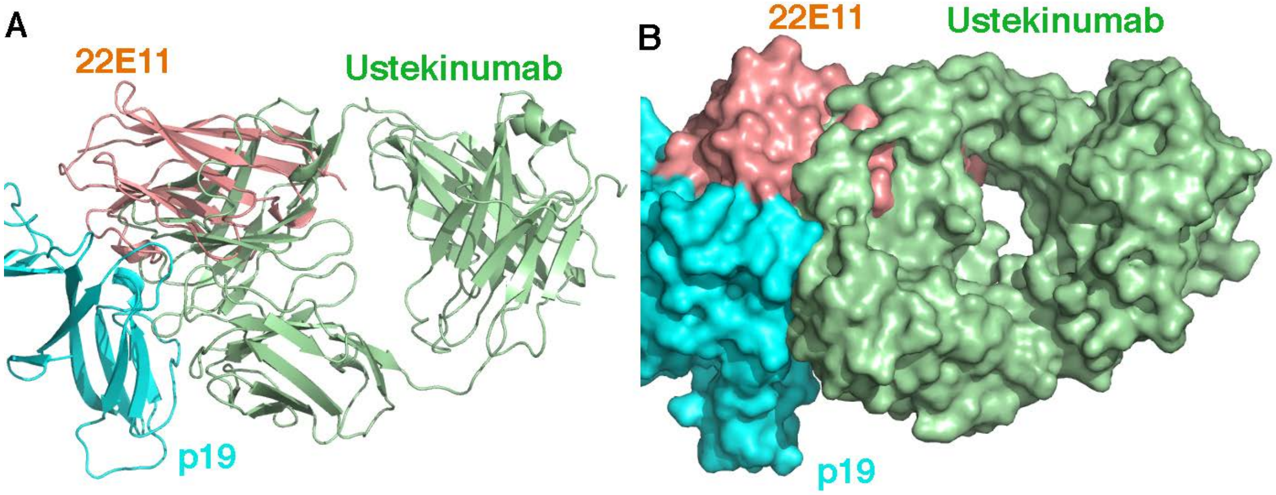

Supplementary figure 6. **Comparison of the superimposed structures of p19 complexes with Nanobody 22E11 and Ustekinumab.** A. Ribbon representation. B. Surface representation. p19 is shown in blue, Ustekinumab in green, and Nb 22E11 is shown in pink.
